# Estimation of dog population in Nasarawa state Nigeria: a pilot study

**DOI:** 10.11604/pamj.2019.34.25.16755

**Published:** 2019-09-12

**Authors:** Ayi Vandi Kwaghe, Daniel Okomah, Ihekerenma Okoli, Mairo Gujba Kachalla, Mohammed Aligana, Olaniran Alabi, Gideon Mbursa Mshelbwala

**Affiliations:** 1 Department of Veterinary and Pest Control Services, Federal Ministry of Agriculture and Rural Development, PMB 135, Area 11, Garki, Abuja, Nigeria

**Keywords:** Pilot study, cross-sectional study, estimated dog population, Nasarawa state, Nigeria

## Abstract

**Introduction:**

Estimation of dog population is relevant in Animal Health Planning; some of the benefits include rabies control and possible elimination, estimation of quantity of dog vaccines and drugs required in the state, policy development and implementation.

**Methods:**

A cross-sectional study was conducted to estimate the population of dogs in Nasarawa state; a local government area (LGA) was randomly selected from each of the three senatorial districts and two wards were selected randomly from the selected LGA's. Three hundred and thirteen questionnaires were administered through face to face interview with dog owners and their dogs in view.

**Results:**

Analysis indicated 97.7% of the dogs were local breeds, 1.7% mixed and 0.3% exotic breeds. Guard dogs were 77% and 23% were used for hunting. Majority of the dogs (67.5%) were owned/stray while 32.5% were owned/confined. In Nasarawa state, 21% of the dogs were vaccinated and 79% had no vaccination history. The low vaccination rate indicates possible threat to animal and human health; hunting dogs are possible source of rabies introduction into their immediate communities from contact with wild reservoirs of the virus. Majority of dogs were between 1-5 years (73%) and more female dogs (52.5%) than males (47.5%) were reported. The dog to household ratio was 1.1:1 while the dog to human ratio is 1.1:6. Estimated number of dogs in Nasarawa state was 462,586 dogs.

**Conclusion:**

Proper sensitization of dog owners on annual antirabies vaccination against rabies in dogs and postexposure prophylaxis in humans is recommended. The local authorities should institute effective measures for the control of stray dogs to prevent the risk of dog bites and other environmental hazards posed by such dogs. The state government should enact and enforce laws on responsible dog ownership to include compulsory annual vaccination of all dogs. This exercise should be replicated in other states of the federation for a comprehensive national dog ecological data necessary for planning, policy development and implementation.

## Introduction

The close relationship between dog and man has existed for several centuries with several advantages and disadvantages [1]. The need for proper identification and management to harness both positive and negative aspects of this relationship is essential for Veterinary Public Health Services in a community [2, 3]. In nearly all parts of the world, dogs pose serious human health, socioeconomic, political, and animal welfare problems [4]. Dogs have been reported to be the principal vectors and reservoirs of rabies in Africa [5]. Rabies is a widespread, neglected and under-reported zoonosis with an almost 100% case fatality rate in humans not treated on time, and causing a significant social and economic burden in many countries worldwide [6]. Domestic dogs are the main reservoir of rabies throughout the developing world [7] enhanced by the persistence of rabies virus in the local dog population and greater movement of infected dogs [8]. More than 90% of all human deaths from rabies occur in the developing world [9]. In Africa and Asia, an estimated 24,000-70,000 people die of rabies each year [10] and the domestic dog is the main source of exposure and primary vector for this important human disease [11]. Mass vaccination of dogs which is also the gold standard is the most successful method for control and possible elimination of dog mediated rabies [12-15]. Also, dogs may harbour a wide range of parasites with zoonotic potential causing health risk to humans [16]. Studies on the use of dogs in Nigeria indicated that people keep dogs for companionship as pet, house guard, assistance for hunting of wildlife and as food animal. Their perceived economic and social worth depends on values attached by the various communities they exist [17].

In industrialized countries, dogs have been trained to remarkably high adaptation to human needs in health promotion; adjustment of the elderly, recovery from illnesses, guiding the blind, assisting the deaf, assisting persons that are impaired in their mobility and to alert epileptic patients that a seizure is imminent, consequently the owner can sit down or take some medications before a seizure strikes [1, 18]. Dogs have also been trained to turn on/off lights, pick up objects and pull wheel chairs for those who are physically challenged. Police dogs have been trained to protect officers, as well as sniffing out drugs, explosives, and other dangerous chemicals beyond what humans can do. Dogs trained for search and rescue missions use their powerful sense of smell to locate people, lost or injured [19]. Studies on dog population are eminent pre-requisite in policy making and planning, for an effective rabies control programme [20]. The population size, ecology, and proportion of ownerless dogs in a community, as well as accessibility of dogs to vaccination campaigns and public attitude towards rabies control programmes are valuable information for planning and evaluation of anti-rabies campaign [20]. The cross-sectional study method that combines direct street count with the administration of questionnaire or door-to-door interview of urban and rural residents is the traditional technique for dog population studies in African countries. This method has been reported as used in Kenya, Madagascar, Nigeria, Tanzania and Zimbabwe by several authors [4, 21-26]. Determination of dog population density (dogs/km^2^) from established indicators of dog abundance (dog to human ratio and dogs per household) is one recommended procedure [17, 27]. The sizes of different segments of a dog population depend heavily on the proportion of human population keeping, tolerating or rejecting dogs in their neighbourhood [19]. The WHO "Guidelines for dog rabies control" has stressed the need for research on dog populations and ecology in urban and rural areas [17]. Lack of reliable estimates of dog populations hampers rabies control campaigns in developing countries, as cost benefit analysis of control strategies cannot be made accurately [17].

## Methods

**Figure 1:**
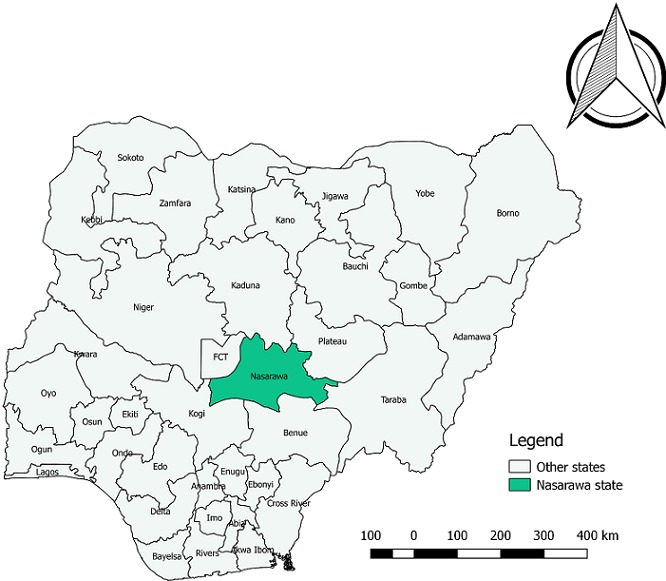
Map of Nigeria indicating Nasarawa state (green colored)

**Figure 2:**
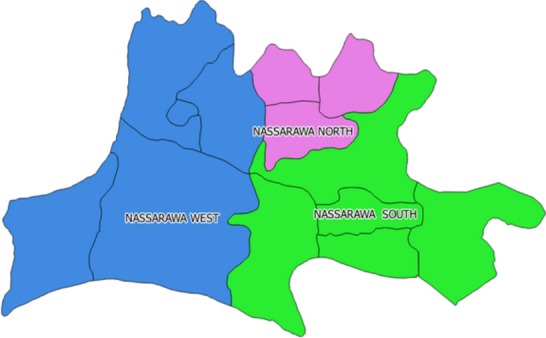
Nasarawa state indicating the three senatorial districts Nasarawa West, Nasarawa North and Nasarawa South

**Study area:** Nasarawa State is bounded in the North by Kaduna State, in the West by the Abuja Federal Capital Territory, in the South by Kogi and Benue States and in the East by Taraba and Plateau States. It has a central location in the Middle Belt region of Nigeria and lies between latitude 7° 45' and 9° 25' N of the equator and between longitude 7° and 9° 37' E of the Greenwich meridian [28, 29]. The state has a total area of 27,117 km^2^ (10,470 sq. mi) and a population of about 1,826,883, according to the 2006 population census. Nasarawa state has 13 local government areas [28, 29]. The map of Nasarawa state and the 3 senatorial districts indicated in Figure 1 and Figure 2.

**Methodology:** a cross-sectional study of the state was designed using multistage sampling method to obtain representative results. Nasarawa state has 13 LGA's, 195 electoral wards in 3 Senatorial districts. Selection of LGA's; One LGA was randomly selected by draw from Nasarawa South (NS), Nasarawa West (NW) and Nasarawa North (NN). These LGA's were selected to cover the three senatorial districts in the state. The LGA's selected were Lafiya with 13 wards (NS) which is the state capital, Keffi has 10 wards (NW) and Nasarawa Eggon with 14 wards (NN). Two wards were selected randomly by draw where the study was conducted. Selected wards include Akurba, Jigdawa, Iya I, Agunji Ogbagi, Angwan Moyi and Gayam. Three hundred and thirteen questionnaires were administered in the six wards.

**Questionnaire design:** the questionnaires that were used for this study was designed by consultants in the field. Section A consisted of enumerator information; name of state, name of location, name of agent, email address, telephone number, date of interview, date of submission and date received. Section B (Animal Information); name, identification mark, breed, purpose, management system, age, sex, source of dog and vaccination history. Section C had animal owner information; name of owner, telephone number, occupation, street address/description and educational status.

**Questionnaire administration:** compound count: this involved house to house administration of questionnaires and dog count. A face interview was conducted by the enumerators for administering the questionnaires to dog owners and leading questions were avoided to eliminate biases while filling the questionnaires. Calculation of average number of people/dogs per household

$$\text{Average number of dogs per house hold (n)=}\frac{\text{Total number of dogs obtained in the study (313) }}{\text{Total number of households covered (281)}}$$

$$\text{Average number of people per house hold (n)=}\frac{\text{Total population of Nasarawa state in 2006 population census (2,523,200)}}{\text{Total number of households in Nasarawa state 2006 population census (420,533)}}$$

**Data analysis:** data was collected and collated. The method of Thrushfield [30] was used in coding the questionnaire. Data was analyzed using the statistical package for the social sciences (SPSS) Version 17.0 and Microsoft Excel. Simple ratio calculations was also used in data analysis.

## Results

Breed types of dogs was determined and at the ward level (Table 1), Jigdawa ward out of the six wards had 4% exotic breed and 96% local breed. Iya I and Akurba wards had mixed breeds of 8% and 2% respectively with the remaining 92% and 98% as local breeds. The remaining three wards; Agunji Ogbagi, Angwan Moyi and Gayam had 100% local breed. At the senatorial district level (Table 2), Keffi (NW) had 4% (mixed breed), 2% (exotic breed) and 94% local breed, Lafiya (NS) had 1% (mixed breed) and 99% (local breed) and 100% (local breed) in Nasarawa Eggon (NN). Nasarawa state (Table 2) had 97.7% Mongrels, 1.7% mixed and 0.7% exotic breeds. Dog breeding in Nasarawa state was identified to have two main purpose; guard and hunting. Analysis indicates that 38% of the dogs in Iya I ward are used for hunting and the remaining 62% as guard dogs (Table 1). In Akurba ward, 100% of the dogs are used for hunting as shown in. The remaining 4 wards; Jigdawa, Agunji Ogbagi, Angwan Moyi and Gayam had 100% guard dogs (Table 1). Result from the various senatorial districts (Table 2); Keffi (NW), had 81% of the dogs as guard dogs and 19% as humting dogs and in Lafiya (NS), 50% each for both hunting and guard dogs while Nasarawa Eggon (NN) had 100% of the dogs as guard dogs. In the entire state (Table 2), 77% are guard dogs while 23% are used for hunting. In the Management systems, most of the dogs (5 out of the six wards) are owned and unconfined (Table 1); Jigdawa, Iya I, Agunji Ogbagi, Angwan Moyi and Gayam had 76%, 88%, 88%, 89% and 64% respectively while the remaining percentages (24%, 12%, 12%, 11% and 36%) are owned and confined respectively. In Akurba ward, 100% of the dogs are owned and confined. Based on the senatorial districts (Table 2), in Keffi (NW), Nasarawa Eggon (NN) and Lafiya (NS); 82%, 88.5% and 64% (owned and unconfined) while 18%, 11.5% and 36% are owned and confined. At the state level, 67.5% (owned and unconfined) while 32.5% are owned and confined (Table 2). Analysis based on vaccination history from the six wards indicates Angwan Moyi had the highest number of vaccinated dogs (41.5%) while Jigdawa, Iya I, Agunji Ogbagi, Akurba and Gayam had vaccinations rates of 6%, 15%, 8%, 20% and 36% accordingly. In all the six wards (Jigdawa, Iya I, Agunji Ogbagi, Angwan Moyi, Akurba and Gayam), majority of the dogs are unvaccinated; 94%, 85%, 92%, 58.5%, 80% and 64% in turn (Table 1). For each of the senatorial districts, Keffi (NW), Nasarawa Eggon (NN) and Lafiya (NS) had vaccination rates of 10.5%, 25% and 28%. Nasarawa state had 21% vaccinated dogs and 79% non vaccination (Table 2).

**Table 1:** Showing percentages of the various categories based on breed, purpose, management system, vaccination history, age, sex and education at ward level in Nasarawa state

Variables	Categories	Wards					
		Jigdawa	Iya I	Agunji Ogbagi	Angwan Moyi	Akurba	Gayam
		Frequency (%)	Frequency (%)	Frequency (%)	Frequency (%)	Frequency (%)	Frequency (%)
**Breed**	Exotic	96.0	92.0	100.0	100.0	98.0	100.0
	Mixed	0.0	8.0	0.0	0.0	2.0	0.0
	Local	4.0	0.0	0.0	0.0	0.0	0.0
	Total	100.0	100.0	100.0	100.0	100.0	100.0
**Purpose**	Hunting	0.0	38.0	0.0	0.0	100.0	0.0
	Guard	100.0	62.0	100.0	100.0	0.0	100.0
	Total	100.0	100.0	100.0	100.0	100.0	100.0
**Management system**	Owned/confined	24.0	12.0	12.0	11.0	100.0	36.0
	Owned/stray	76.0	88.0	88.0	89.0	0.0	64.0
	Total	100.0	100.0	100.0	100.0	100.0	100.0
**Vaccination history**	Vaccinated	6.0	15.0	8.0	41.5	20.0	36.0
	Not vaccinated	94.0	85.0	92.0	58.5	80.0	64.0
	Total	100.0	100.0	100.0	100.0	100.0	100.0
**Age**	<1 year	46.0	16.7	16.0	13.0	0.0	22.0
	1-5 years	48.0	71.7	76.0	87.0	82.0	74.0
	>5 years	6.0	11.7	8.0	0.0	18.0	4.0
	Total	100.0	100.0	100.0	100.0	100.0	100.0
**Sex**	Male	54.0	50.0	24.0	49.0	58.0	50.0
	Female	46.0	50.0	76.0	51.0	42.0	50.0
	Total	100.0	100.0	100.0	100.0	100.0	100.0
**Education**	Primary	28.0	20.0	40.0	47.2	98.0	40.0
	Secondary	54.0	51.7	44.0	28.3	0.0	26.0
	Tertiary	18.0	28.3	16.0	24.5	2.0	34.0
	Total	100.0	100.0	100.0	100.0	100.0	100.0

**Table 2:** Showing percentages of the various categories based on breed, purpose, management system, vaccination history, age, sex and education at senatorial district level and in Nasarawa state

VARIABLES	CATEGORIES	SENATORIAL DISTRICTS			STATE
		KEFFI (NW)	NASARAWA EGGON (NN)	LAFIYA (NS)	
		Frequency (%)	Frequency (%)	Frequency (%)	Frequency (%)
**Breed**	Exotic	2.0	0.0	0.0	0.7
	Mixed	4.0	0.0	1.0	1.7
	Local	94.0	100.0	99.0	97.7
	Total	100.0	100.0	100.0	100.0
**Purpose**	Hunting	19.0	0.0	50.0	23.0
	Guard	81.0	100.0	50.0	77.0
	Total	100.0	100.0	100.0	100.0
**Management system**	Owned/confined	18.0	11.5	68.0	32.5
	Owned/stray	82.0	88.5	32.0	67.5
	Total	100.0	100.0	100.0	100.0
**Vaccination history**	Vaccinated	10.5	25.0	28.0	21.0
	Not vaccinated	89.5	75.0	72.0	79.0
	Total	100.0	100.0	100.0	100.0
**Age**	<1 year	31.4	14.5	11.0	19.0
	1-5 years	59.8	81.5	78.0	73.0
	>5 years	8.8	4.0	11.0	8.0
	Total	100.0	100.0	100.0	100.0
**Sex**	Male	52.0	36.5	54.0	47.5
	Female	48.0	63.5	46.0	52.5
	Total	100.0	100.0	100.0	100.0
**Education**	Primary	24.0	43.6	69.0	52.2
	Secondary	52.9	36.2	13.0	34.0
	Tertiary	23.1	20.2	18.0	20.5
	Total	100.0	100.0	100.0	100.0

Majority of the dogs were between 1-5 years of age (reproductive age). Jigdawa (48%), Iya I (71.7%), Agunji Ogbagi (76%), Angwan Moyi (87%), Akurba (82%) and Gayam (74%). Age ranges below a year were; Jigdawa (46%), Iya I (16.7%), Agunji Ogbagi (16%), Angwan Moyi (13%), Akurba (0%) and Gayam (22%) while the age ranges above 5 years were; Jigdawa (6%), Iya I (11.7%), Agunji Ogbagi (8%), Angwan Moyi (0%), Akurba (18%) and Gayam (4%) (Table 1). Similar results were exibited at the senatorial districts; majority of the dogs (1 to 5 years), Keffi (NW) (59.8%), Nasarawa Eggon (NN) (81.5%) and Lafiya (NS) (78%); age ranges that were less than a year were 31.4%, 14.5% and 11% and those above 5 years were 8.8%, 4% and 11% respectively (Table 2). At the state level, 19% (< 1 year), 73% (1-5 years) and 8% (> 5 years) (Table 2). Iya I and Gayam wards had equal number of both sexes (50% males and 50% females) each while in Jigdawa and Akurba wards,the number of male dogs (54% and 58%) were more than that of females (46% and 42%). In Agunji Ogbagi and Angwan Moyi, the percentage of female dogs (76% and 51%) were more than that of the males (24% and 49%) respectively (Table 1). Two out of the three senatorial districts (Keffi and Lafiya) had more male dogs (52% and 54%) than female dogs (48% and 46%) (Table 2). Nasarawa Eggon (NN) had more females (63.5%) as compared to males (36.5%) (Figure 1). In general, Nasarawa state had more female dogs (52.5%) than males (47.5%) (Table 1). There were more people with primary education at Gayam (40%), Akurba (98%) and Angwan Moyi (47%). Other results for primary education were Agunji Ogbaji (40%), Iya I (20%) and Jigwada (28%). Secondary eduction was more at Agunji Ogbaji (44%), Iya I (51.7%) and Jigwada (54%). Others were, Gayam (26%), Akurba (0%) and Angwan Moyi (28.3%). Tetiary education in all the wards was low; Gayam (34%), Akurba (2%), Angwan Moyi (24.5%), Agunji Ogbaji (16%), Iya I (28.3%) and Jigwada (18%). At the senatorial district, primary education was highest at lafiya (NS) with 69%, 43.6% at Nasarawa Eggon (NN) and 24% at Keffi (NW) (Table 1). Secondary Education; 52.9% Keffi, 36.2% Nasarawa Eggon and 13% Lafiya (Table 2). Highest level of tetiary eduction was at Keffi (23.1%), Nasarawa Eggon with 20.2% and 18% for Lafiya. Nasarawa state had 45.5% primary education, 34% secondary education and 20.5% tetiary education (Table 2). Sources from which the dogs were brought from indicates that majority of the dogs were from Nasarawa state (94.3%), Kano (5.1%) and Kaduna (0.3%) states while Abuja had 0.3%. Out of the 313 dogs that were covered in 281 households, the dog:house hold ratio was 1.1:1 and the dog:human ratio was 1.1:6. Hence the estimated number of dogs in Nasarawa state from this pilot study was 462,586 dogs.

## Discussion

Over 95% of the dogs in the state were local breeds with few mixed and exotic breeds. None of the dogs were treated as pets or reared for breeding in this study indicating the need for a dog breeding programme in the state to boost the production of mixed and exotic breeds of dogs in the state. Similarly, report from Niger state [31] revealed majority of the dogs 60.1% as native (local), 14.5% mixed and 25.4% exotic breeds. Also, in agreement with this study is the report from Lagos state [32] where majority of the dogs were local breeds (41.9%), 29.5% crossbreeds (mixed) and 25.5% exotic breeds. Contrary to what was obtained in this study were the studies from Ilorin [19], where 35.4% of the dogs were exotic breeds, 30% local breeds and 34.6% cross (mixed breed) and in Aba, Abia state where 66.3% of the dogs were exotic breeds, 19% local breed and 14.7% cross (mixed) breed [33]. The high percentage of local breed of dogs in Nasarawa state could be closely associated with the purpose of dog breeding in the state; mainly for guard and hunting and the wards where the exercise was conducted were mostly local settings. Nasarawa state had 77% guard dogs and 23% hunting dogs in agreement with the report from Ilorin, Kwara state [19] where guard dogs were the majority (49.4%) and the remaining were kept for various purposes. Similarly, in Aba, majority of the dogs (63.5%) were used as guard dogs [33]. In Akurba ward, all the dogs were used for the purpose of hunting which also relates to the danger of the dogs cotracting rabies from the wild and bringing it to their immediate community. Also, Iya I ward, had quite a number of hunting dogs and are liable to similar danger as Akurba ward. This danger is also connected to the high level of non-vaccination in the state; the entire state had 21% vaccination rate, implying that 79% of the dogs in Nasarawa state have never been vaccinated regardless of the type of vaccine that was used. Angwan Moyi ward happens to be the only ward that had up to 40% vaccination history (antirabies vaccination), the rest of the wards had very low vaccination rates. The issue of dog vaccination especially antirabies vaccination need to be taken seriously in the state.

There is serious need for public enlightenment on rabies in the state and to relate the need for annual antirabies vaccination for their dogs. From other studies so far conducted in other states, Nasarawa state had the least vaccination rate of dogs against rabies; 39.9% in Aba vaccinated against rabies [33], 64.1% dogs vaccinated in Lagos [32] and in Niger state, 69.6% were vaccinated [31]. The percentage of dogs vaccinated in this study is far below the World Health Organization standard of vaccination of 70-80% dog population in an area in order to boost herd immunity [34]. This reveals the immense need for sensitization in Nasarawa state (antirabies vaccination campaign) as compared to other states of the Federation where similar studies were conducted such as in Lagos [32] and Niger states [31], where the percentage of dogs vaccinated against rabies almost met the WHO requirement for rabies control. In connection with the purpose of dog rearing and low antirabies vaccination, is the issue of the management systems of the dogs in the state where majority are owned and unconfined (semi-intensive system of management); they roam about around the vicinity and are fed at their various homes. This type of pratice is not good as it relates to the control of rabies in the state. The sensization earlier on mentioned should include confinement of dogs by owners for safe keeping and elimination of public health threats. In this study, 67.5% of the dogs in Nasarawa state are owned and unconfined while 32.5% are owned and confined, similar to a report where intensive management (kennel confined) were 26.1% and extensive (free-roaming) were 73.7% [19].

The age of dogs by percentage in Nasarawa state is cartegorized as 19% (< 1 year), 73% (1-5 years) and 8% (> 5 years) implying that most of the dogs are between 1-5 years of age in their reproductive age. There was absence of pupies in Akurba ward (< 1 year of age) which is relevant for the continuation of dog population in the community. The community need to be enlightened in this area so that the importance of raising puppies in the ward will be taken seriously and implemented. Similar finding was reported where 32.8% of the dogs were below one 1 year of age, 42.3% (1-5 years) and 24.9% (>5years) [31]. In another study, 31.72% of dogs in Lagos state were less than 1 year old and 68.28 % >1 year [32]. The study revealed female dogs (52.5%) in the state compared to the males (47.5%). Similar reports in Lagos state where more female dogs (59.71%) than males (40.29%) where obtained [32]. This is contrary to report from Niger state where 65% of the dog population were males and 35% were females [31] and in Aba, Abia state where 62.2% were males and 37.8% were female dogs [33]. The dog:household ratio was 1.1:1 while the dog:human ratio was 1.1:6 which is quite high compared to other related studies. This study concurs with several findings 1:5.4 in Niger state [31] and 1:5.6 in Lagos state [32]. Contrary to the low density of dogs in other urban cities in Nigeria where dog to human ratio was 1:43 in Makurdi, Benue state [35] and 1:1000 in Moslem dominated part of Kaduna North, Kaduna state [17]. The dog:human ratio most commonly lies between 1:6 and 1:10, but considerable variation exists [17]. Urban areas in Africa have a ratio of 1:21.2, while rural areas in Africa have a ratio of 1:7.4 [10]. Other studies around the world who reported dog: human ratios similar to what was obtained in this study include 1:4.6 in Kenya, Brazil and Thailand [24, 36, 37] and 1:4.3 in Mexicali [38]. Studies elsewhere around the globe that differ from this study include 1:14 in Tanzania [39]; Maboloko, Bophuthatswana 1:11 [40], in the peri-urban Kikambuani, Kenya, 1:15 [24], in sub-urban Zimbabwe 1:16 [22], in N'Djaména, the capital of Chad 1:21.5 [41] and in Mutendere, Zambia, 1:45 [42]. The ratio of owned dogs to people is usually higher in rural areas of a country, but there is also considerable variation within cities [13]. The structure and turnover of a dog population are determined by a great number of different factors. Its analysis depends on vital statistics such as sex and age ratios, natality and rearing success, and survival and mortality rates [43].

## Conclusion

The dog to house hold ratio was 1.1:1, while the dog to human ratio was 1.1:6. Estimated number of dogs in Nasarawa was 462,586. There was a very low vaccination rate of dogs in Nasarawa state. There is the need by the Local Authority to institute effective measures for the control of dog movements (management systems) to prevent the risk of dog bites and other environmental hazards posed by such dogs. The State Government should enact and enforce laws on responsible dog ownership to include compulsory annual vaccination of all dogs. This exercise should be replicated in other States of the Federation as soon as possible in order to have a comprehensive National Dog Ecological data necessary for planning and policy development.

### What is known about this topic

- Population of dogs have been estimated in a number of studies in various states in Nigeria;
- Estimation of dog population is used in animal health planning such as in rabies control;
- Such studies also stipulate the uses of dogs in a particular area of study.

### What this study adds

- The study has provided us with the estimated number of dogs in Nasarawa state which can be used in animal health planning in the state especially rabies control in dogs;
- Data obtained indicates low vaccination rate of dogs in Nasarawa state hence the need for sensitization of dog owners to indulge in antirabies vaccination of their dogs for their safety and the health of the general public;
- The study provided data on dog: household ratio (1.1:1) in the state.

## Competing interests

The authors declare no competing interests.
